# Genomic Diversity of SARS-CoV-2 Omicron Variant in South American Countries

**DOI:** 10.3390/v14061234

**Published:** 2022-06-07

**Authors:** Nicolas Luna, Marina Muñoz, Angie L. Ramírez, Luz H. Patiño, Sergio Andres Castañeda, Nathalia Ballesteros, Juan David Ramírez

**Affiliations:** 1 Centro de Investigaciones en Microbiología y Biotecnología—UR (CIMBIUR), Facultad de Ciencias Naturales, Universidad del Rosario, Bogotá 111221, Colombia; nicolas.luna@urosario.edu.co (N.L.); claudia.munoz@urosario.edu.co (M.M.); angiel.ramirez@urosario.edu.co (A.L.R.); lhpatinoblan@yahoo.com (L.H.P.); sergio.castaneda@urosario.edu.co (S.A.C.); nathalia.ballesteros@urosario.edu.co (N.B.); 2 Molecular Microbiology Laboratory, Department of Pathology, Molecular and Cell-Based Medicine, Icahn School of Medicine at Mount Sinai, New York, NY 10029, USA

**Keywords:** SARS-CoV-2, Omicron sublineages, South America, nucleotide diversity, phylogenomic analysis

## Abstract

Genomic surveillance of SARS-CoV-2 is one of the tools that provide genomic information on circulating variants. Given the recent emergence of the Omicron (B.1.1.529) variant, this tool has provided data about this lineage’s genomic and epidemiological characteristics. However, in South America, this variant’s arrival and genomic diversity are scarcely known. Therefore, this study determined the genomic diversity and phylogenetic relationships of 21,615 Omicron genomes available in public databases. We found that in South America, BA.1 (n = 15,449, 71%) and BA.1.1 (n = 6257, 29%) are the dominant sublineages, with several mutations that favor transmission and antibody evasion. In addition, these lineages showed cryptic transmission arriving on the continent in late September 2021. This event may have contributed to the dispersal of Omicron sublineages and the acquisition of new mutations. Considering the genomic and epidemiological characteristics of these lineages, especially those with a high number of mutations in their genome, it is important to conduct studies and surveillance on the dynamics of these lineages to identify the mechanisms of mutation acquisition and their impact on public health.

## 1. Introduction

Genomic surveillance is one of the tools that provide real-time information on the circulating variants of SARS-CoV-2. This information allows understanding of its genomic diversity, dispersal, and transmission mechanisms [1]. Additionally, together with public data, it allows for characterizing and dating different lineages that impact public health and/or can modulate the spread of the virus. Particularly with variants of concern (VOCs), which have higher transmissibility and virulence rates [1,2], genomic surveillance has provided insight into the genomic characteristics and global diversity of these variants [3,4].

Recently, a new emerging lineage (B.1.1.529) has spread globally, causing an increase in the number of COVID-19 cases. This VOC, known as Omicron, harbors a high number of mutations that favor immune evasion, increased transmissibility and decreased vaccine efficiency in preventing infection [5,6]. Moreover, this variant has shown several dispersals and global transmission events [7] associated with the different Omicron sublineages (BA.1, BA.1.1, BA.2 and BA.3). Compared to the sublineages of other VOCs (i.e., Delta), these have unique genomic characteristics that have generated a public health impact [7]. Therefore, surveillance and genomic studies have focused on analyzing these characteristics that have enabled Omicron to create a significant health turmoil.

Despite the vast number of studies about the surveillance of Omicron in some regions around the world, the genomic diversity and introduction date of this variant into South American countries remain unknown. Therefore, this study aimed to characterize the diversity of Omicron sublineages circulating in South America using comparative genomics of public genomes available for the region.

## 2. Materials and Methods

We analyzed 21,615 high-quality and complete Omicron genomes from South America available in the GISAID database until 28 February 2022 (Table S1). The analysis of these genomes was performed according to the Muñoz et al. (2021) and Ramírez et al. (2020) schemes [7,8]. These schemes consisted of SARS-CoV-2 lineage typing using the PANGOLIN-v1.9 tool (https://github.com/cov-lineages/pangolin (accessed on 10 May 2022)), and alignment and phylogenetic analysis using the NextClade tool v1.5.4 default command line (https://docs.nextstrain.org/projects/nextclade/en/stable/user/nextclade-cli.html (accessed on 10 May 2022)), which performs sequence alignment, variant calling, clade assignment and maximum-likelihood (ML) tree placement. On the other hand, this considers genome georeferencing and mutational analysis of SARS-CoV-2 coding regions.

Subsequently, we estimated the potential introduction date and dispersion dynamics of Omicron into the continent using TreeTime software [9], which considers a fixed clock rate of 0.8 × 10^−3^ (SD = 0.4 × 10^−3^) [10], a strict clock (SC) under a coalescent tree skyline prior and a root step to minimize residuals in a root-to-tip. For this analysis, we used the alignment and ML tree from Nexclade, a dataset with 2365 reference genomes belonging to other SARS-CoV-2 lineages (Table S1) available from the phylogenomic dataset of auspice.us (https://auspice.us/ (accessed on 10 May 2022)) and the collection from genomes analyzed to obtain an ML time-scaled phylogeny. Six iterations were run during the TreeTime analysis, and the marginal date estimates of ancestral states were inferred with 95% confidence intervals (95% CI).

## 3. Results

**Figure 1:**
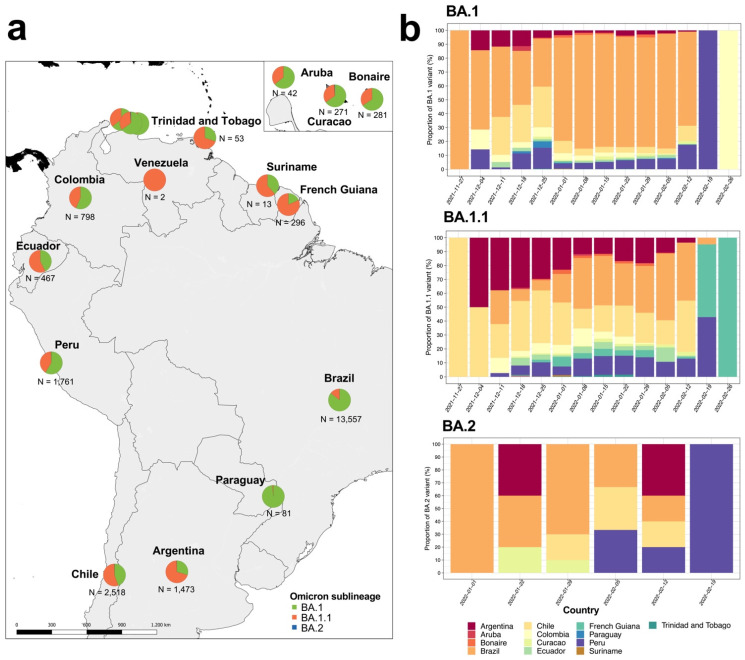
Descriptive analysis of Omicron sublineages circulating in South America. (**a**) The geographical distribution, proportion and number of Omicron genomes for each country. (**b**) Temporal variations in the proportions of Omicron sublineages reported for each country.

We analyzed 21,615 Omicron genome assemblies from 14 South American countries (Table S1). These genomes corresponded to three Omicron main sublineages (BA.1, BA.1.1 and BA.2), with abundances that varied across South American countries (Figure 1a). The BA.1 was significantly predominant in four countries: Brazil (n = 11,618; 85.6%), Colombia (n = 452; 56.6%), Paraguay (n = 80; 98.8%) and Peru (n = 1033; 58.7%) (Table S2 and Figure 1b). Meanwhile, BA.1.1 was significantly predominant in five countries: Argentina (n = 1034; 70.1%), Chile (n = 1448; 55.2%), Ecuador (n = 268; 57.4%), French Guiana (n = 242; 81.8%), and Trinidad and Tobago (n = 37; 69.8%) (Table S2 and Figure 1b). As for BA.2, we found few genomes reported on the continent; hence the abundance of this sublineage was not significant (Table S2). On the other hand, we found variations in the proportions of Omicron reports from each country by lineage (Figure 1b), where BA.1 and BA.1.1 were first reported in late November 2021 in Brazil and Chile, while BA.2 was first reported in early January 2022 in Brazil.

**Figure 2:**
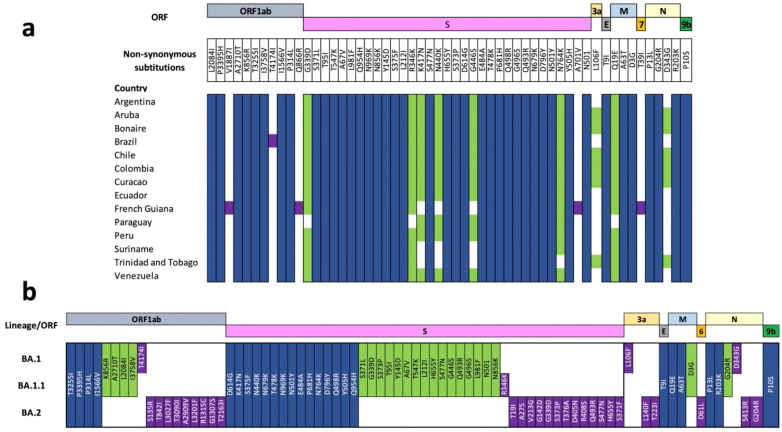
Nucleotide diversity of analyzed Omicron genomes. The figure represents the non-synonymous amino acid substitutions found in the 21,615 SARS-CoV-2 whole-genome sequences compared with the Wuhan reference sequence (NC_045512.2). (**a**) Mutational analysis between South American countries. (**b**) Mutational analysis between Omicron sublineages (BA.1, BA.1.1 and BA.2). For both analyses, substitutions found in more than 10% of the genomes analyzed were considered. The blue color represents the substitutions shared between the 21,615 genomes analyzed, the green color represents the substitutions found in the genomes of some countries, and the purple color represents the substitutions considered unique for each country.

The mutational analysis showed fifty-five non-synonymous amino acid substitutions (Figure 2a). Forty-one of these substitutions (75%) were shared, with seven present in at least eleven countries and two substitutions in six countries. Additionally, we identified four unique substitutions in French Guiana and one in 15% of the genomes from Brazil (Figure 2a). When analyzing the different Omicron sublineages, we observed twenty-five substitutions shared among sublineages (Figure 2b), most of them in the Spike gene. Furthermore, twenty-two of these shared substitutions were found between BA.1 and BA.1.1, with sixteen (72%) located in the Spike. Finally, we found thirty-one unique non-synonymous substitutions, twenty-seven of them (87%) identified only in BA.2, mostly found in the Spike (Figure 2b).

**Figure 3:**
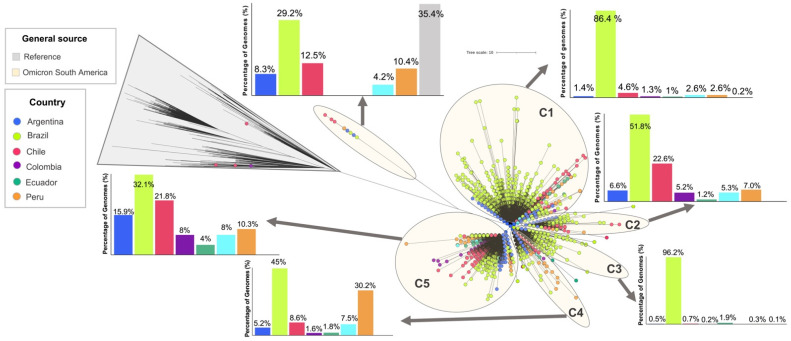
Phylogenetic analysis of Omicron genomes circulating in South America. The figure shows the ML tree with the phylogenomic relationships and the abundance of genomes by geographical origin. Countries with genome abundance greater than 400 were described, while countries with abundance less than that threshold were included in the “other countries” category (Cyan).

The Omicron genomes analyzed in this study were clustered into five main monophyletic clusters labeled C1–C5 (Figure 3) plus a minor divergent cluster. The minor divergent cluster included 48 genomes that corresponded mostly to reference genomes (n = 17; 35.4%) and genomes from Brazil (n = 14; 29.2%) belonging to BA.2 (Table S3). This cluster was the only one that included the analyzed genomes of this sublineage (Table S3). Clusters C1 and C3 were predominantly constituted by genomes from Brazil (96.2% and 86.4%, respectively) with a minimal proportion from other South American countries. The genomes of these clusters consisted predominantly of BA.1 with an abundance of more than 50% (Table S3). In the case of clusters C2, C4 and C5, Brazil remained in first place with the most abundant genomes, but the distribution was more homogeneous, with an increased abundance in countries such as Chile (C2 and C5), Peru (C4) and Argentina (C5). These clusters were mainly composed of sublineages BA.1.15 (n = 1139; 67.9%), BA.1 (n = 2244; 98%) and BA.1.1 (n = 5500; 78.7%) respectively (Table S3). Although these clusters had a higher abundance of BA.1 and BA.1.1, they had divergent lineage genomes from these sublineages (Table S3).

The time-scaled phylogeny obtained from TreeTime showed the introduction date based on a node-date assignment from the most recent common ancestor (MRCA) (Figure S1). In general, the estimated arrival date for the Omicron variant in South America was at the end of September 2021 (Table S4 and Figure S1), while the introduction of the pruned clades containing C1 sequences was September 29, 2021 (95% CI = 11 September 2021 to 13 October 2021). The putative introduction date of clusters circulating in South America is described in Supplementary Table S5. These findings suggest that the Omicron variant was circulating in Brazil, subsequently spreading to other South American countries such as Chile, Peru and Argentina by mid-October and November 2021.

## 4. Discussion

Genomic monitoring of SARS-CoV-2 variants in different regions of the world allows understanding of the dynamics and genetic mechanisms of each variant [1], especially in Omicron, that has several mechanisms to promote increased transmission and immune escape [7,11]. Furthermore, this variant has an estimated transmission rate between 5 and 8 [12], which means that Omicron is transmitted rapidly, leading to an increase in the number of associated cases. In South America, we found that BA.1, BA.1.1 and BA.2 are circulating across the continent, and the first two are predominant in particular countries (Figure 1b). These lineages are characterized by a high number of mutations in the genome that facilitate rapid transmission and evasion of the immune system [5]; most are in the Spike gene [11]. The analyzed genomes of these sublineages have various mutations of epidemiological interest (S373P, G446S, S477N, N679K and L981F) (Figure 2b), involved in the transmission and escape of antibodies [13]. That would imply an impact on the transmission dynamics of the virus on the continent. Despite the dramatic increase in reported cases under vaccination schedules, there is no increase in the number of deaths or hospitalizations [14]. Thus, future studies should focus on these dynamics considering the vaccination programs and genomic characteristics of Omicron in each country.

As for BA.2, it is interesting to note the low number of genomes reported so far and the circulation in only a few countries on the continent (Table S1 and Figure 1b) and the minor divergent cluster (Figure 3). Unlike BA.1 and BA.1.1, this lineage has a greater number of unique mutations, mainly in the Spike gene, promoting more effective immune evasion and transmissibility [7,8,9,10,11,12,13]. Moreover, this number of unique mutations might be contributing to the associated divergent clustering for this lineage, which differs from the other clusters that contain the BA.1 and BA.1.1 genomes. Although, to date, the circulation of the BA.3 lineage, that has similar genomic characteristics with BA.1 and BA.1.1 [7], has not been reported. It might be circulating in the continent in the future and have a similar impact as BA.1 and BA.1.1. Considering the characteristics and public health impact of the Omicron sublineages, especially BA.2, in the future, these lineages might be circulating and dispersing on the continent. Therefore, genomic and epidemiological studies are needed to monitor their dynamics in the region.

The emergence of Omicron in South America might be explained by dispersal pathways, such as international flights, facilitating the transmission and circulation of SARS-CoV-2 between countries [2,15], hence the arrival of new variants. In addition, the characteristics of Omicron may have facilitated the rapid spread across the continent [16]. The introduction seems to have occurred in Brazil at the end of September. However, this finding contrasts with that reported in Nextstrain (7 September 2021 in Guyana (CI = 8 June 2021 to 16 October 2021)) (https://nextstrain.org/ncov/gisaid/south-america/all-time (accessed on 10 May 2022)) and is more recent than the first case in the continent (24 November 2021) (Table S5) [17]. Following its arrival on the continent, Omicron spread to other countries through its transmission mechanisms between October and December (Tables S4 and S5) while acquiring mutations considered unique to each geographical location at the amino acid level (Figure 2a). Therefore, considering the genomic characteristics and the recent reports of BA.2, this sublineage might have emerged in December 2021.

Despite obtaining information in terms of the relationships and evolutionary times of the genomes analyzed from the continent, the dataset studied includes countries with few high-quality genomes available until the end of February 2022. Therefore, the variability in the number of genomes available in each country might affect the phylogenetic results, especially when analyzing dispersal events between countries and comparing them with real-time data from other genomic analysis programs. Nevertheless, this available data provides relevant information on the emergence and possible clustering of sublineages in South America. Therefore, in the future, to analyze dispersal dates and Omicron assemblages in each of the countries, unique and representative genomes of these geographic areas are needed.

The genomic landscape of Omicron in South America provides mutational and phylogenetic information about circulating lineages. Previous studies have reported similar results with sublineages of other VOCs in the region, where the circulation of these sublineages in diverse regions of the continent has favored the acquisition of different genomic characteristics which might affect the molecular diagnosis such as the emergence of new lineages [3,4]. Furthermore, genetic variation, especially in the amino acids, might generate impacts on public health [3]. Herein, we found unique and shared mutations by sublineage and country (Figure 2), most of which are found in the spike gene. This might be related to vaccination rates per country, promoting the acquisition of new mutations [18]. However, future studies are needed to determine the impact of vaccination schedules on the genomic structure of Omicron.

In conclusion, South America is mainly dominated by the BA.1 and BA.1.1 lineages, which emerged from Brazil at the end of September 2021. Furthermore, at the genomic level, these sublineages present unique and/or shared mutations, which might affect molecular diagnosis, transmissibility and even favor the emergence of new sublineages. Therefore, future studies and surveillance should focus on the current genomic dynamics of Omicron sublineages and their potential impact on public health in South American countries.

## Data Availability

Not applicable.

## Supplementary Materials

The following supporting information can be downloaded at: https://www.mdpi.com/article/10.3390/v14061234/s1, Table S1: Metadata of Omicron lineages (BA.1, BA.1.1 and BA.2) genomes from South America analyzed in this work: Omicron genomes from South America (n = 21,615) and SARS-CoV-2 lineages reference genomes (n = 2365); Table S2: Number of Omicron lineage genomes reported for each country. Table S3: Number of genomes of the Omicron sublineages in each cluster obtained from the phylogenetic analysis. Table S4: Dates of the introduction of Omicron’s circulating clusters to the South American continent. Table S5: Dates of the first Omicron genome sequenced for each South American country analyzed. Figure S1: Maximum-likelihood (ML) time-scaled phylogeny. This figure highlights with a diamond the approximate date of Omicron’s emergence in South America (29 September 2021).
