# Supplementary figures and images for: Genomic Diversity of SARS-CoV-2 Omicron Variant in South American Countries
Source: Viruses. 2022 Jun 7;14(6):1234. doi: 10.3390/v14061234 (PMC9230695; doi:10.3390/v14061234)

Tree scale: 0.001

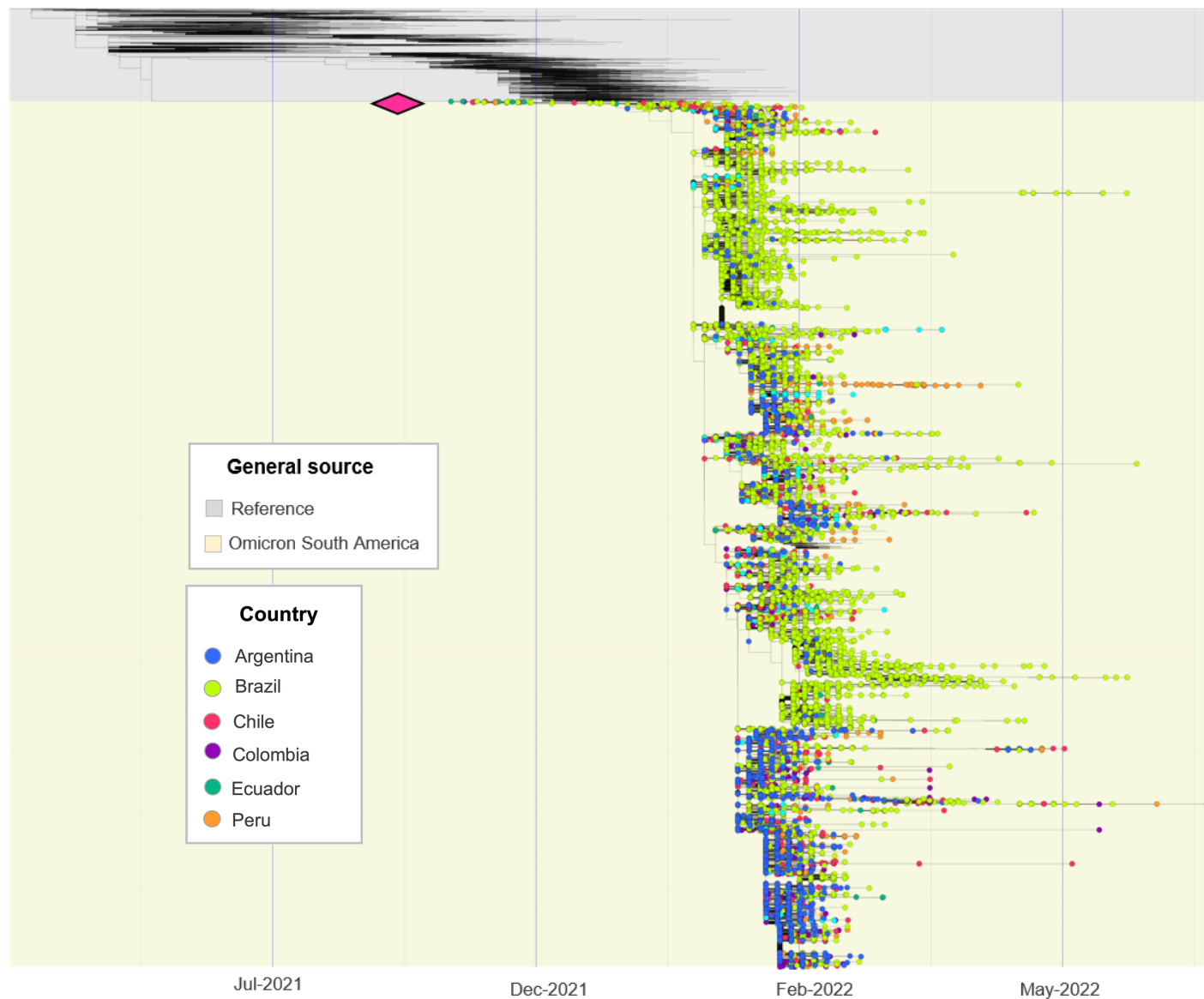

Supplement: Supplementary file 1 [file viruses-14-01234-s001.zip › Figure S1.pdf]
